# Holey MoS_2_ Nanosheets with Photocatalytic Metal Rich Edges by Ambient Electrospray Deposition for Solar Water Disinfection

**DOI:** 10.1002/gch2.201800052

**Published:** 2018-09-10

**Authors:** Depanjan Sarkar, Biswajit Mondal, Anirban Som, Swathy Jakka Ravindran, Sourav Kanti Jana, C. K. Manju, Thalappil Pradeep

**Affiliations:** ^1^ DST Unit of Nanoscience (DST UNS) and Thematic Unit of Excellence (TUE) Department of Chemistry Indian Institute of Technology Madras Chennai 600036 India

**Keywords:** chemical drilling, holey‐MoS_2_ NSs, Mo‐rich edges, nanometer size holes, water disinfection

## Abstract

A new method for creating nanopores in single‐layer molybdenum disulfide (MoS_2_) nanosheets (NSs) by the electrospray deposition of silver ions on a water suspension of the former is introduced. Electrospray‐deposited silver ions react with the MoS_2_ NSs at the liquid–air interface, resulting in Ag_2_S nanoparticles which enter the solution, leaving the NSs with holes of 3–5 nm diameter. Specific reaction with the S of MoS_2_ NSs leads to Mo‐rich edges. Such Mo‐rich defects are highly efficient for the generation of active oxygen species such as H_2_O_2_ under visible light which causes efficient disinfection of water. 10^5^ times higher efficiency in disinfection for the holey MoS_2_ NSs in comparison to normal MoS_2_ NSs is shown. Experiments are performed with multiple bacterial strains and a virus strain, demonstrating the utility of the method for practical applications. A conceptual prototype is also presented.

2D sheets derived from MX_2_, where M is a metal and X represents a chalcogen (S, Se, Te), exhibit interesting properties.^1^ Examples include optoelectronics,^2^ catalysis,^3^, ^4^ hydrogen evolution,^3^, ^5^ biomolecules detection,^6^ and lithium‐ion storage.^7^ Both theoretical^8^ and experimental^9^ studies have shown that the edges are more active catalytically than the basal plane. Hence, defect‐rich MoS_2_ NSs are of more interest to researchers due to their increased effective surface area.^10^ Creating such defects in a single‐layer MoS_2_ sheet is a challenge. Defects can be created by atom and electron bombardment, which requires sophisticated instrumentation. In the recent past, we have developed a methodology for creating functional nanomaterials under ambient conditions which requires no sophisticated instrumentation.^11^ In this paper, we have demonstrated a method to create defect‐rich 2D nanosheets for applications like solar disinfection of water.

Drinking water scarcity is one of the most alarming problems of the modern world. Rapid, energy‐efficient desalination and disinfection processes are required to address the problem.^12^ 2D nanomaterials like graphene are shown to be efficient materials for water purification. There are several reports where graphene‐based materials were used for the removal of arsenic,^13^ salts,^14^ dyes,^15^ antibiotics,^16^ pesticides,^17^ etc. A few recent reports show that MoS_2_ NSs have potential utility in water purification. A recent computational study shows that MoS_2_ NSs with nanopores can effectively desalinate water.^18^ They have also shown that water flux is higher (70% higher than the graphene nanopores) for a hole with Mo‐rich edges.^18^ In another very recent report, vertically aligned MoS_2_ NSs were shown to be effective for disinfection of water under visible light.^19^ The reactive edges of the NSs are responsible for the generation of active oxygen species like H_2_O_2_ which, in turn, is the cause of disinfection.^19^ Hence, vertical alignment of the MoS_2_ NSs was made for the enhancement of reactive areas. These reports prove the tremendous potential of defect‐rich MoS_2_ NSs.

Here we report an easy, cost‐effective, ambient, solution‐based method to create nanopores in single‐layer MoS_2_ NSs. In this process, Ag^+^ ions are electrosprayed on a water suspension of chemically synthesized MoS_2_ NSs. In the course of the deposition, Ag^+^ ions react with the NSs and form Ag_2_S, leaving the former with Mo‐rich defects. The size of the holes can be controlled by varying the deposition time. We show that these nanoporous MoS_2_ NSs are highly effective in water disinfection under visible light. A prototype device based on such holey MoS_2_ has been demonstrated illustrating the applicability of such methods in field conditions.

When a high potential in the range 2–2.5 kV was applied to the nano electrospray ionization (nESI) source of 15–20 µm diameter (at the tip), through a platinum (Pt) wire electrode (0.1 mm), filled with aqueous solution of silver acetate (AgOAc), a spray plume of solvated Ag^+^ ions was observed at the source. This plume was directed toward the surface of the grounded aqueous suspension of MoS_2_ NSs. **Figure** **1** shows the chemical drilling process of MoS_2_ NSs schematically where the spherical pink balls represent the solvated Ag^+^ ions. The mass spectrum collected from the spray plume (Figure S1, Supporting Information) confirms the presence of Ag^+^ and [Ag(H_2_O)]^+^ ions. Ag^+^ ions react with the NSs and form Ag_2_S NPs. With time, Ag_2_S NPs go into water resulting in nanoporous MoS_2_ NSs. The whole process is demonstrated schematically in Figure 1 where 2D MoS_2_ NS is shown using blue (represents Mo) and yellow (represents S) balls. The experimental procedure discussed in the Supporting Information. After the process of drilling, the resultant holey MoS_2_ NS formed is also shown in Figure 1 which shows Mo‐rich edges at the defect positions. This has been proved by high‐angle annular dark‐field transmission electron microscopy (HAADF TEM) imaging (explained in detail at a later part of the paper). X‐ray photoelectron spectroscopic (XPS) measurements were also performed to support this result. The Mo 3d region after electrospray deposition (ESD) of silver showed a blueshift of 0.3 eV in binding energy corresponding to a decrease in number of sulfide ions compared to the parent material (Figure S2, Supporting Information). A detailed description has been provided in the Supporting Information. The reaction of Ag^+^ ions with MoS_2_ NSs in the solution phase was also supported by a recent report from our group.^20^ To prove that the deposition of ions followed by the reaction is the only reason for the defects in MoS_2_ NSs and discard any possibility of pre‐existing defects in the as‐synthesized NSs, detailed characterization of the as‐synthesized MoS_2_ NSs was performed using various spectroscopic and microscopic techniques. Figure S3a (Supporting Information) shows TEM image of the as‐synthesized MoS_2_ NSs. From the TEM images, it is seen that with our synthesis, we have obtained thin single‐layer sheets of MoS_2_ with nanometer dimension. From the scanning transmission electron microscopic (STEM) image (Figure S3b, Supporting Information) it is clear that the sheets are single crystalline in nature and do not have defects. Raman spectroscopic measurement of the as‐synthesized NSs was also done to further confirm the quality of the NSs. Figure S3c (Supporting Information) shows the Raman spectra collected from MoS_2_ NSs (red trace) and MoS_2_ bulk (black trace), respectively. Peaks at 386 cm^−1^(E_2g_) and 404 cm^−1^ (A_1g_) prove the 2D nature of the NSs. Increase in the full width at half maximum (FWHM) for A_1g_ mode and a decrease in the peak difference of A_1g_ and E_2g_ modes indicate the successful exfoliation of bulk MoS_2_ to the 2D nanoscale form.^21^ UV–vis spectrum collected from the aqueous suspension of MoS_2_ NSs also shows the characteristic peaks at 410, 620, and 672 nm (Figure S3d, Supporting Information). Electrospray deposition experiments were performed with these sheets. The deposition time and rate were optimized. In a typical experiment, Ag^+^ ions were electrosprayed on a suspension of MoS_2_ NSs (3.7 × 10^−3^ m with respect to Mo) for 30 min at a deposition current of 60 nA. After the deposition, a portion of the NSs was seen floating on the water surface and the rest of it was in the bulk. Both of these categories of NSs were taken on a carbon‐coated TEM grid for imaging. **Figure** **2**a shows a STEM image of the as‐synthesized MoS_2_ NS. The high‐resolution TEM (HRTEM) image (collected from the floating layer of NSs) in Figure 2b shows the presence of holes (indicated with red circles) in a MoS_2_ NS. The HRTEM image was taken from a single‐layer MoS_2_ NS to prove the clear discontinuity of the lattice planes. Figure 2c shows a STEM image of MoS_2_ NSs after the creation of holes. The image clearly shows part of a hole in a single‐crystalline NS present in bulk water. The dimensions of the holes were in the range of 3–5 nm. We speculate dynamics of the suspended NSs on the liquid surface during deposition leading to the creation of holes in all the NSs. This motion can be due to the transfer of charged droplets on the water surface in presence of a tangential electric field. From the independent experiments, we know that hydroxyl ions (due to their high mobility), generated due to hydrolysis of water, are the charge carriers from the center of the spray to the ground electrode kept at the rim of the liquid reservoir, causing a hydrodynamic flow in the liquid.^22^ This flow may be the reason for the presence of nanoporous sheets both on the surface of the water and in the bulk. To see the fate of the NSs with longer deposition time, an experiment was performed where the deposition time was kept at 2 h. Figure S4a (Supporting Information) shows a TEM image taken from the suspension after the experiment. In this case, we see that the whole NS was reacted and got converted to Ag_2_S NPs. Figure S4b (Supporting Information) shows an HRTEM image of a single Ag_2_S NP. This experiment also proves that Ag^+^ ions react with MoS_2_ NS and go to the solution as Ag_2_S NPs along with a portion of Mo from the reacted region, which subsequently becomes MoO_4_^2−^.^20^ The process of the selective reaction of incoming Ag^+^ ions makes the holey MoS_2_ NSs porous with Mo‐rich edges. HAADF STEM image shown in Figure 2d clearly shows the Mo enriched edges of the holes in a single‐layer MoS_2_ NS. From Figure 2d, the distance between the two Mo atoms is 0.26 nm. Considering the holes as a circle of 3 nm diameter, the number of Mo atoms per hole is ≈35. Thus, due to hole formation, a 1 µm × 1 µm NS exposes ≈2.7 × 10^5^ additional Mo atoms. Note that the STEM image of Figure 2c suggests >7800 holes µm^−2^. This process of creating nanoscale holes on 2D sheets by ambient ion reactions is referred to as chemical drilling. We have maintained low deposition time in order to control the size of the holes. For this reason, in all our experiments of creating nm sized holes in the MoS_2_ NSs (except the control experiment showing longer deposition leading to complete reaction of the NSs to form Ag_2_S NPs), the concentration of the Ag_2_S NPs formed was negligible in comparison to the amount of MoS_2_ in solution.

**Figure 1:**
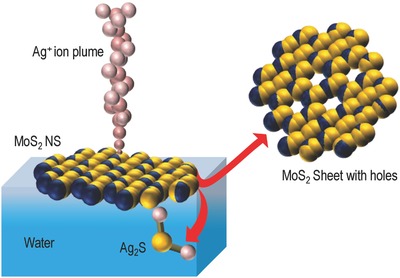
Drilling of MoS_2_ NSs by ambient ions. Schematic representation of chemical drilling of MoS_2_ NSs using electrospray‐deposited Ag^+^ ions. Not to scale.

**Figure 2:**
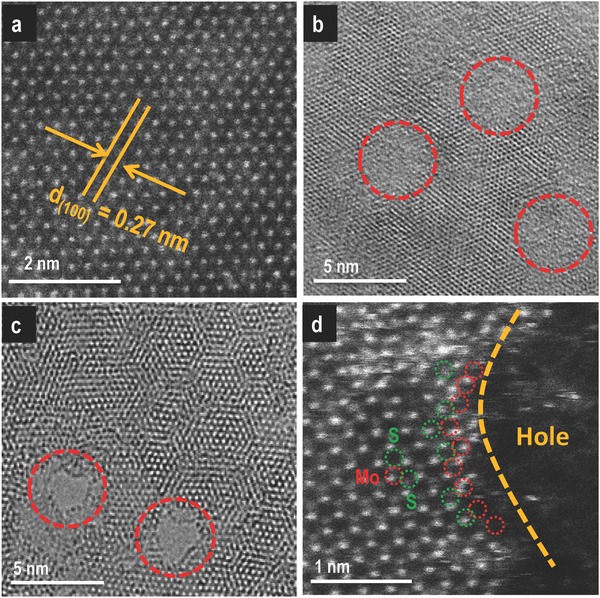
Characterization of holey MoS_2_ NSs. a) STEM image of the as‐synthesized MoS_2_ NSs showing no defects in it. b) HRTEM image of a MoS_2_ NS, floating on water after the deposition process, with holes. c) STEM image of the same showing clear holes on a single NS. d) HAADF STEM image of a holey MoS_2_ NS taken from the subphase showing a Mo enriched edge. Red circles in image (d) denote Mo and the green circles represent S.

To check the efficiency of disinfection of holey MoS_2_ NSs, we have performed a series of experiments along with several control experiments. We have tested the disinfection efficiency of the MoS_2_ NSs using bacteria and virus‐contaminated water. **Figure** **3**a–d shows optical photographs of the live bacteria observed after disinfection by pour plate technique. The inset shows magnified images in each case where Figure 3a–d represents input concentration (diluted two times), disinfection experiment with bulk MoS_2_, disinfection with the as‐synthesized MoS_2_ NSs, and disinfection with holey MoS_2_ NSs, respectively. The contaminated water when exposed to porous MoS_2_ NSs and visible light (Supporting Information) showed 10 000 000 times reduction in bacteria, from 10^7^ to 10^0^ CFU mL^−1^ for *Escherichia coli* (or 7 log reduction), in 2 h whereas the as‐synthesized MoS_2_ NSs and bulk MoS_2_ with equivalent concentration of Ag, used for the drilling process, showed only 1% of disinfection efficiency (or 2 log reduction) (Figure 3e). But holey MoS_2_ NSs in dark showed only 3 log reduction (Figure 3e) in the bacteria count. We note that Ag^+^ at a concentration above 50 ppb can be an excellent disinfectant.^23^ Under the same experimental conditions, bulk MoS_2_ showed negligible disinfection efficiency for the mentioned bacterial input. This suggests that chemical drilling makes the MoS_2_ nanosheets photocatalytically more active. We speculate that the Mo‐rich defects in the NSs provide enhanced active surface area for the generation of reactive oxygen species (ROS). The disinfection efficiency of the material was also tested with gram‐positive bacteria (*Bacillus subtilis*). It was noticed that *B. subtilis* are more resistive toward H_2_O_2_ than *E. coli* under the same experimental conditions (Figure S5, Supporting Information).

**Figure 3:**
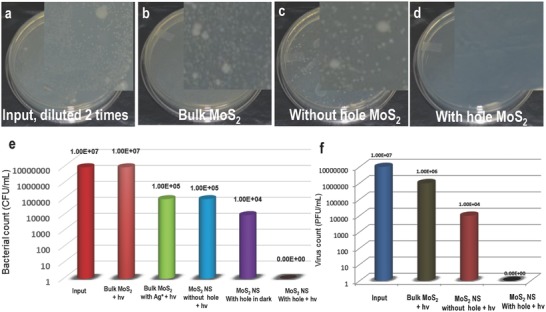
Disinfection performance of holey MoS_2_ NSs. a–d) Optical photographs of the live bacteria observed after disinfection by pour plate technique. Inset shows magnified images in each case. a) Input concentration, diluted two times, b) disinfection experiment with bulk MoS_2_, c) disinfection with the as‐synthesized MoS_2_ NSs, and d) disinfection with holey MoS_2_ NSs. Comparison of the e) antibacterial and f) antiviral activity of the porous MoS_2_ NSs, with Mo‐rich edges. The activity is compared with bulk MoS_2_, bulk MoS_2_ with the same amount of Ag used for drilling, MoS_2_ NSs without holes, and holey MoS_2_ NSs in dark. The test organisms used was F‐specific bacteriophage MS2 grown on *E. coli* (ATCC 15597‐B1). In all the experiments, parameters such as light intensity, irradiation time (2 h), sample concentrations, etc., were maintained constant. The entire visible spectrum was exposed to the sample although one frequency (ν) is mentioned.

In the following part of the paper, we have shown that holey MoS_2_ NSs are more efficient in generating at least one ROS species, namely, H_2_O_2_ (see later). The disinfection efficiency of Ag^+^ alone was also examined under the same experimental conditions taking the same amount of Ag^+^ ions used for chemical drilling. Ag^+^ ions showed a negligible effect on *E. coli* inactivation because the concentration of Ag^+^ was 0.2 × 10^−6^ m, much lower than the concentration needed for disinfection at a bacterial load of 10^7^ CFU mL^−1^.

Considering the fact that the major water purification techniques used for virus removal are the addition of chlorine which produces harmful disinfection byproducts, we propose that the holey MoS_2_ NSs could serve as an efficient method to reduce pathogenic viruses by a safer method. We observed that while bulk MoS_2_ with and without Ag^+^ and MoS_2_ NSs without holes were unable to affect the phage significantly (Figure S6, Supporting Information), the porous MoS_2_ NSs were found to achieve a 7 log reduction by photocatalytic disinfection (Figure 3f). Moreover, by this study, we proved that this method can disinfect virus at comparatively higher concentrations.

From earlier reports, we know that MoS_2_ in presence of visible light can generate active oxygen species like H_2_O_2_.^19^ Liu et al. have shown that the edges of MoS_2_ NSs are more active in this reaction. We believe that generation of H_2_O_2_, in presence of visible light is one of the reasons for the disinfection of water. To prove that the holey MoS_2_ is more efficient for the production of H_2_O_2_, a set of cyclic voltammetry (CV) experiments were performed. For all our CV experiments, we have used a precleaned gold electrode as the working electrode, Ag/AgCl was used as the reference electrode, and Pt was used as the counter electrode. Prior to CV measurements of each sample, 5 mL of as‐synthesized MoS_2_ suspension was dried at 55 °C in a glass bottle. Subsequently, CV experiments were performed by adding 5 mL of 1 m phosphate buffered saline (PBS, pH ≈ 7.3) to the bottle having previously dried MoS_2_ and electrochemical experiment was performed after exposing the dispersion to visible light (for 1 or 2 h, depending on the experiment). CV of each sample was performed from 0 to +1 V with a fixed scan rate of 100 mV s^−1^. CV profiles of Au, performed in blank solution (only PBS), as well as in PBS along with as‐synthesized MoS_2_ NSs and holey MoS_2_ NSs irradiated with visible light for 1 and 2 h, respectively, are shown in **Figure** **4**. Each CV profile has two major peaks, one observed around +0.95 V corresponds to the formation of AuCl_4_^−^ during the forward potential scan (0 to +1 V) and another around +0.43 V due to the reduction of gold chloride in the reverse potential scan (+1 V to 0). Along with these, a small hump was observed in the CV scan, around +0.45 V (marked portion in the spectrum was multiplied three times for better visualization) which corresponds to the oxidation of H_2_O_2_. In order to gain a deeper insight into the sensing mechanism, we have performed CV measurements in different electrolytes like aqueous PBS, NaCl, and Na_2_SO_4_ and these traces are shown in Figure S7 (Supporting Information). From such experiments, we concluded that the peak at +0.45 V is due to electrooxidation of H_2_O_2_.^24^ In the course of the reaction, M—OH or M=O might be formed as intermediates which ultimately get converted to ROS. Although the reaction mixture with as‐synthesized MoS_2_ NSs shows some amount of H_2_O_2_, the holey MoS_2_ NSs are more efficient in the generation of H_2_O_2_ because of the presence of enhanced reactive surface area. Oxidation of H_2_O_2_ by Au electrode was further confirmed by a control experiment (CV trace shown in Figure S8, Supporting Information) performed in PBS in presence of externally added H_2_O_2_ at different concentrations. To prove the fact that defects in the MoS_2_ sheets have a significant role in H_2_O_2_ generation, a control experiment was carried out. In this experiment, two sets of holey MoS_2_ samples were synthesized by varying the deposition time of Ag^+^ (10 and 20 min at a deposition rate of 100 nA). More deposition time will enable the creation of more holes in NSs. All the other parameters were kept constant and the CV was measured successively. Figure S9 (Supporting Information) shows the feature corresponding to H_2_O_2_ in the voltammogram. The H_2_O_2_ concentration was higher in the second sample, that is, the MoS_2_ NSs with more holes (Ag deposition for 20 min). To quantify the H_2_O_2_ generated from holey MoS_2_, we have performed CV measurements (traces shown in Figure S8, Supporting Information) in PBS with successive addition of commercial H_2_O_2_ at different concentrations (0.4 × 10^−6^ to 1 × 10^−3^ m). The oxidation current as a function of H_2_O_2_ concentration (Figure 4b) shows a linear behavior. Oxidation current of H_2_O_2_ (data from Figure 4a have been shown in the inset of Figure 4b after background subtraction) produced by holey MoS_2_ was compared with the calibration curve (Figure 4b), giving a quantitative measurement of H_2_O_2_ produced by various materials (Table S1, Supporting Information). We note that in the disinfection process other ROS species may also be involved, although we have detected only H_2_O_2_.

**Figure 4:**
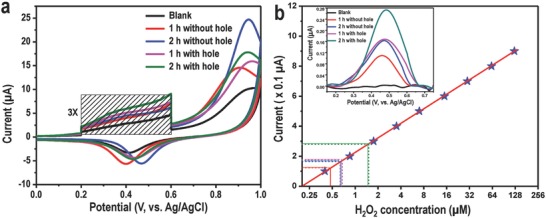
Quantitative detection of H_2_O_2_ using CV. a) Cyclic voltammetry (CV) traces of 5 mL of MoS_2_ solution, in phosphate buffered saline (PBS), using a gold electrode. b) Quantification of H_2_O_2_ concentration generated by holey MoS_2_ NSs and a calibration curve which is extracted from Figure S7a (Supporting Information) (H_2_O_2_ oxidation current as a function of externally added H_2_O_2_ concentration, measured with Au electrode in PBS solution). The intercepts marked with red, blue, violet, and green lines are due to MoS_2_ nanosheets without hole after 1 h, MoS_2_ nanosheets without hole after 2 h, holey MoS_2_ after 1 h, and holey MoS_2_ after 2 h, respectively. Inset shows background corrected CV traces of holey MoS_2_ and MoS_2_ NSs without hole (derived from data in (a)).

**Figure** **5**a shows the disinfection process schematically. Scanning electron microscopy (SEM) imaging establishes the damage of bacterial cells. Figure 5b shows the SEM image of the bacterial cells before disinfection. Here, we see healthy cells of *E*. c*oli*. On the other hand, Figure 5c,d shows the SEM images of dead *E*. c*oli* cells at different magnifications. It is seen clearly from these images that the bacterial cells are damaged (red circles) due to reaction with H_2_O_2_. Similar cell damage was observed when the bacteria were treated with commercially available H_2_O_2_ (Figure S10, Supporting Information). Dark field fluorescence imaging of bacteria was also performed to prove 100% disinfection. Fluorescence staining experiments using dark field microscopy can distinguish between intact and membrane‐permeable cells using LIVE/DEAD Baclight bacterial viability kit. The two stains used were the membrane impermeant propidium iodide (PI), causing red fluorescence of membrane permeabilized cells and SYTO 9, a nucleic acid binding green fluorescent dye that could enter into all the cells regardless of whether they were intact or permeabilized. PI fluorescing defective bacteria were observed as red (Figure 5f) when they were treated with holey MoS_2_ nanosheets (and irradiated) whereas untreated (and irradiated) bacteria show SYTO 9 emission (green) (Figure 5e). Membrane permeabilization is seen in all the bacteria upon treatment with holey MoS_2_ nanosheets proving 100% disinfection. TEM images of the virus before (Figure 5g–i) and after (Figure 5h–j) irradiation show a clear contrast difference. This is because the uranyl acetate stain enters defective viral capsids and causes a distinct difference in contrast between untreated and treated viruses. Dense dark centered capsids (defective capsids) which had taken up the uranyl acetate stain were seen as dark whereas untreated viruses were rigid and did not show the stain within.^23^ All the above disinfection processes were performed under a Xenon arc lamp.

**Figure 5:**
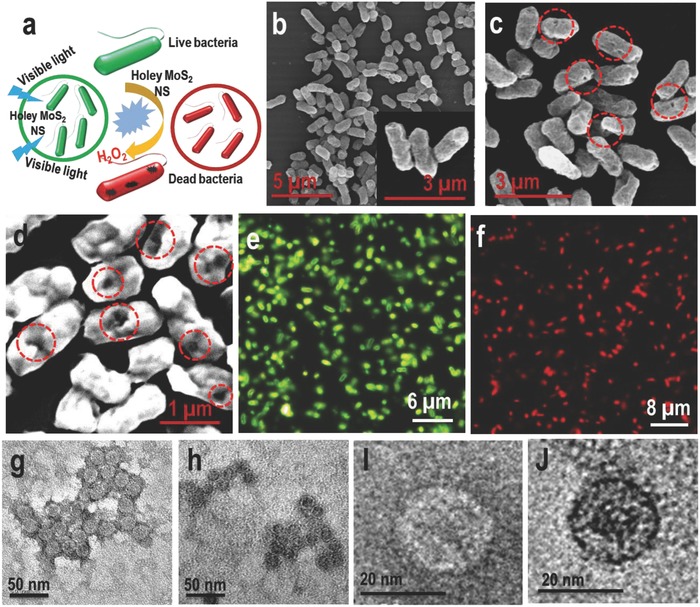
Imaging of bacteria and virus before and after the treatment with the samples. a) Schematic representation of the disinfection process; green and red colors represent live and dead cells, respectively. b) SEM image of *E. coli* cells before disinfection. Inset shows an SEM image of *E. coli* cells at higher magnification. c,d) SEM image of *E. coli* cells after disinfection. Red circles show the damage in the cells due to reaction with H_2_O_2_. e,f) Dark field fluorescence microscopic analysis of live and dead bacteria cells showing 100% disinfection after treating with holey MoS_2_ NSs under visible light. g,h) TEM images of virus before (live virus) and after (dead virus) the treatment with the sample (holey MoS_2_), respectively. i,j) Magnified TEM images of live and dead viruses show the clear contrast difference between the two.

We also have designed a prototype for the disinfection of water using a commercially available low power light‐emitting diode (LED) strip. **Figure** **6**a shows the schematic of a prototype where holey MoS_2_ NSs supported on alumina were packed in a borosilicate glass tube (column size and radius were 10 in. and 3 mm inner diameter, respectively) and a LED (4.8 W m^−1^) strip was wrapped around it. Contaminated water was pushed from below using a syringe pump and pure water was collected from the top. Antigravity flow of the contaminated water was chosen for longer contact of it with the holey MoS_2_ NSs. We have used a bacterial load of 10^3^ CFU mL^−1^ in this case. Contaminated water was passed through the column multiple times and after each cycle, the sample was taken for plating (Figure S11, Supporting Information). We see 100% disinfection after five cycles (Figure 6b). Leaching of the MoS_2_ NSs from the alumina support was checked using UV–vis spectroscopy and inductively coupled plasma mass spectrometry (ICP‐MS) measurements (Figure S12, Supporting Information). No significant leaching was observed in ICP‐MS studies. A control experiment was also performed where the column was packed with alumina and contaminated water was passed through it under the same experimental conditions mentioned above. No difference in the bacterial count was noticed between the input and output of contaminated water (Figure S13, Supporting Information).

**Figure 6:**
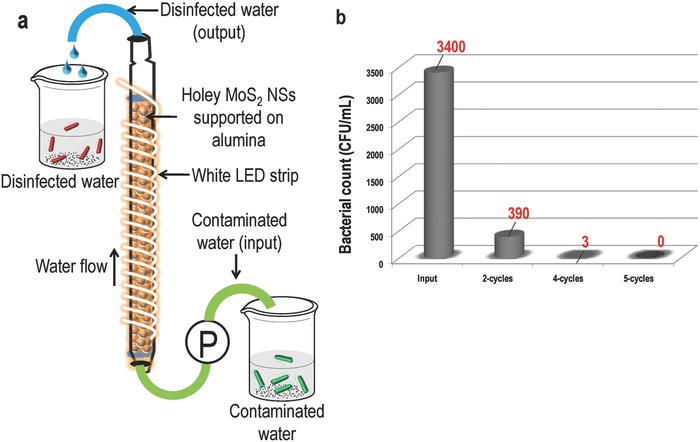
Disinfection performance holey MoS_2_ using a prototype. a) Prototype for water disinfection using low power LED strips and b) plot of bacterial count after 2–5 cycles of operation.

An inexpensive ambient method was demonstrated for making nanometer‐sized holes in MoS_2_ NSs composed of molybdenum‐rich edges. Immobilized porous MoS_2_ NSs in water make H_2_O_2_ upon exposure of visible light and disinfect microbially contaminated water samples. The composition provides antibacterial and antiviral activity at concentrations of 10^7^ CFU mL^−1^ bacteria and 10^7^ PFU mL^−1^ virus. Mo‐rich edges of MoS_2_ NSs are more catalytically active toward the generation of H_2_O_2_ which in turn reacts with the bacterial and viral cells and damage them. We also show a working prototype using immobilized nanosheets on oxide supports for water disinfection using low‐power LEDs. The simplicity of the process and use of very low amount of metal make this material a potential candidate for practical applications.

## Experimental Section

For testing disinfection, 5 mL of synthetic water (typically containing *E. coli* ATCC 25922 at a concentration of 1 × 10^7^ CFU mL^−1^, unless otherwise mentioned) was treated with 3.7 × 10^−3^ m of MoS_2_ suspension (in terms of Mo) of porous MoS_2_ nanosheets and the photocatalytic disinfection performance was checked under visible light. Xenon lamp equipped with UV filter was used as the light source. The disinfection efficiency was compared with the controls, namely, (a) equal mass of bulk MoS_2_, (b) same concentration of Ag^+^ used for chemical drilling along with bulk MoS_2_, and (c) as‐synthesized MoS_2_ nanosheets, all under visible light, at the same experimental conditions. A high concentration of the bacterial input was maintained considering the activity of the proposed material in the treatment of water from challenging environments. About 1 mL of the diluted samples was plated along with nutrient agar on a sterile Petri dish using the pour plate method after 2 h of reaction time. After 48 h of incubation at 37 °C, the colonies were counted and recorded.

In the case of antiviral testing, ≈1 × 10^5^ bacteriophage MS2 was used. The prepared synthetic water was shaken gently with the 3.7 × 10^−3^ m of MoS_2_ suspension (in terms of Mo) of porous MoS_2_ nanosheets and left for a contact time of 2 h for the photocatalytic disinfection and subsequently the viable virus count was measured by double‐layer plaque assay (using *E. coli* host). The plates were incubated for a period of 20–24 h at 37 °C.

All other experimental details including synthesis of MoS_2_ NS, electrospray deposition on MoS_2_ NSs, bacteria and virus used for disinfection reaction, and dark field fluorescence microscopic analysis are available in the Supporting information.

## Conflict of Interest

The authors declare no conflict of interest.

## Supporting information

Supplementary
