# Supplementary material for: Holey MoS2 Nanosheets with Photocatalytic Metal Rich Edges by Ambient Electrospray Deposition for Solar Water Disinfection
Source: Glob Chall. 2018 Sep 10;2(12):1800052. doi: 10.1002/gch2.201800052 (PMC6607134; doi:10.1002/gch2.201800052)
Supplement: Supplementary file 1 — Supplementary [file GCH2-2-1800052-s001.pdf]

Copyright WILEY-VCH Verlag GmbH & Co. KGaA, 69469 Weinheim, Germany, 2018.

Global Challenges
Open Access

Supporting Information

for *Global Challenges,* DOI: 10.1002/gch2.201800052

Holey MoS_2 Nanosheets with Photocatalytic Metal Rich Edges by Ambient Electrospray Deposition for Solar Water Disinfection

*Depanjan Sarkar, Biswajit Mondal, Anirban Som, Swathy Jakka Ravindran, Sourav Kanti Jana, C. K. Manju, and Thalappil Pradeep**

Supporting Information

Holey MoS_2 Nanosheets with Photocatalytic Metal Rich Edges by Ambient Electrospray Deposition for Solar Water Disinfection

*Depanjan Sarkar,† Biswajit Mondal,† Anirban Som, Swathy Jakka Ravindran, Sourav Kanti Jana, C. K. Manju and Thalappil Pradeep**

EXPERIMENTAL SECTION

All the commercially available chemicals were used as is, without further purification. Silver acetate (AgOAc), molybdenum disulfide (MoS_2), 1.6 M n-butyllithium in hexane and the solvent, hexane were purchased from Sigma Aldrich, India.

Synthesis of MoS_2 NS. Chemical exfoliation method was used to synthesize MoS_2 NSs from MoS_2 powder.[1] 300 mg of MoS_2 powder was taken in a round bottom flask, under an argon atmosphere, and 3 mL of 1.6 M n-butyllithium was added. The resulting solution was stirred for 2 days under the same conditions. Then the resulting lithium intercalated product was washed repeatedly with hexane, to remove excess reactants, followed by the addition of 100 mL of distilled water. The produced was sonicated in a bath sonicator for 1 h. This aqueous dispersion of MoS_2 NSs was centrifuged at a speed of 18000 rpm to remove un-exfoliated MoS_2. The quality of the synthesized MoS_2 NSs was checked using electron microscopy, UV-Vis and Raman spectroscopy. The concentration of MoS_2 dispersion was determined using inductively coupled plasma mass spectrometry (ICP MS).

Electrospray deposition on MoS_2 NSs. For electrospray deposition, a home built nanoelectrospray ionization (nESI) source was used. The nESI source was made by pulling a borosilicate glass capillary (0.86 mm ID and 1.5 mm OD) into two, using a micropipette puller (Sutter Instruments, U.S.A.). Each tip, after pulling, was checked using an optical microscope to ensure the size and quality of the cut. Tips with an opening of 10-15 µm were

used for all the deposition experiments. 10 mM aqueous solution of AgOAc was filled in the nESI tips using a micro injector pipette tip and Pt wire was inserted into the solution, making an electrode for high voltage connection. For electrospray deposition on MoS_2 NSs, an aqueous suspension of 3.7 mM (in terms of Mo) MoS_2 NS was taken in an Eppendorf vial and the Ag^+ ions generated by the nESI source was guided towards it. The distance between the tip of the nESI source and the surface of the MoS_2 solution was optimized to be 10 mm. No changes in the size or nature of the holes were observed with change in the distance between the tip and the deposition surface provided the amount of the ions were constant. The water MoS_2 suspension was grounded through a picoammeter using a copper strip. The deposition current was varied from 20-100 nA for different experiments.

Details of the bacteria and virus used for disinfection reaction. *Escherichia coli* and MS2 bacteriophage, the surrogates for water borne pathogens (bacteria and viruses, respectively) were used for antimicrobial testing. Saline water condition was used for all the antibacterial and antiviral testing. A fresh single colony of the *Escherichia coli* (ATCC 10536) pre-grown on Luria–Bertani (LB) agar was used as the inoculum and cell suspensions in LB broth were allowed to grow at 37 °C to late exponential phase with a final optical density (600 nm) of 1. This was used as the input for antibacterial experiments. In the case of antiviral testing, F-specific bacteriophage MS2 (ATCC 15597-B1) was cultured using *E. coli host C-3000* (ATCC 15597) in the logarithmic phase. Purified virus was used as input for antiviral experiments.

Electrochemical measurements.

Both cyclic voltammetry (CV) and linear sweep voltammetry (LSV) were measured by an electrochemical analyzer (CHI 600A) with conventional three-electrode configuration adopted with bulk Au (111) electrode as the working electrode, silver/silver chloride (Ag/AgCl) as the reference electrode and Pt wire as the counter electrode. Prior to any electrochemical measurement, Au electrode was cleaned manually with two different micro

polishing powders of Al_2O_3 (particle size ~ 0.3 µM and 0.05 µM). All the electrochemical measurements were performed at room temperature and in phosphate buffered saline (Merck India Pvt. Ltd) solution of pH~ 7.3.

Dark field fluorescence microscopic analysis. Fluorescence microscopy imaging was performed using a Cytoviva microscopy system. For sample preparation, LIVE/DEAD Baclight^{TM} bacterial viability kit (Molecular Probes, Eugene, OR) was used. At each time point, 1 mL of the sample (holey MoS_2 nanosheet treated and control bacteria) was mixed with 2 µL of PI-SYTO 9 mix (1:1) and incubated in dark for 15 min. About 0.5–1 µL sample was spotted on a 1 mm thick ultrasonically cleaned glass slide (SCHOTT) and it was covered with a 0.145 mm thick cleaned glass cover slip (SCHOTT). Imaging was performed using 100X oil (Cargille) immersion objective.

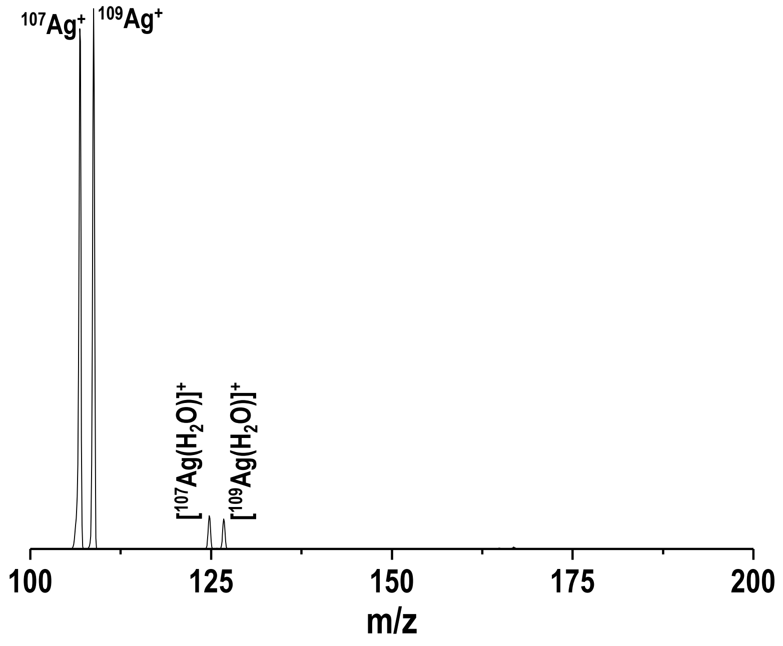

Figure S1. nESI of AgOAc**.** Mass spectrum collected from the nESI of AgOAc.

The Mo 3d region of parent materials in XPS showed two peaks at 229.5 and at 232 6 eV binding energy corresponding to $3d_{5/2}$ and $3d_{3/2}$ of Mo(IV). But when the XPS spectrum after the electrospray of Ag^+ ions was taken, a blue shift of 0.3 eV was seen due to the decrease in

a number of sulphide ions (because of the reaction with silver). The emergence of two new peaks is attributed to the formation of Mo(VI)O_4^{2-} (as a result of the Mo sites lost due to reaction).[20]

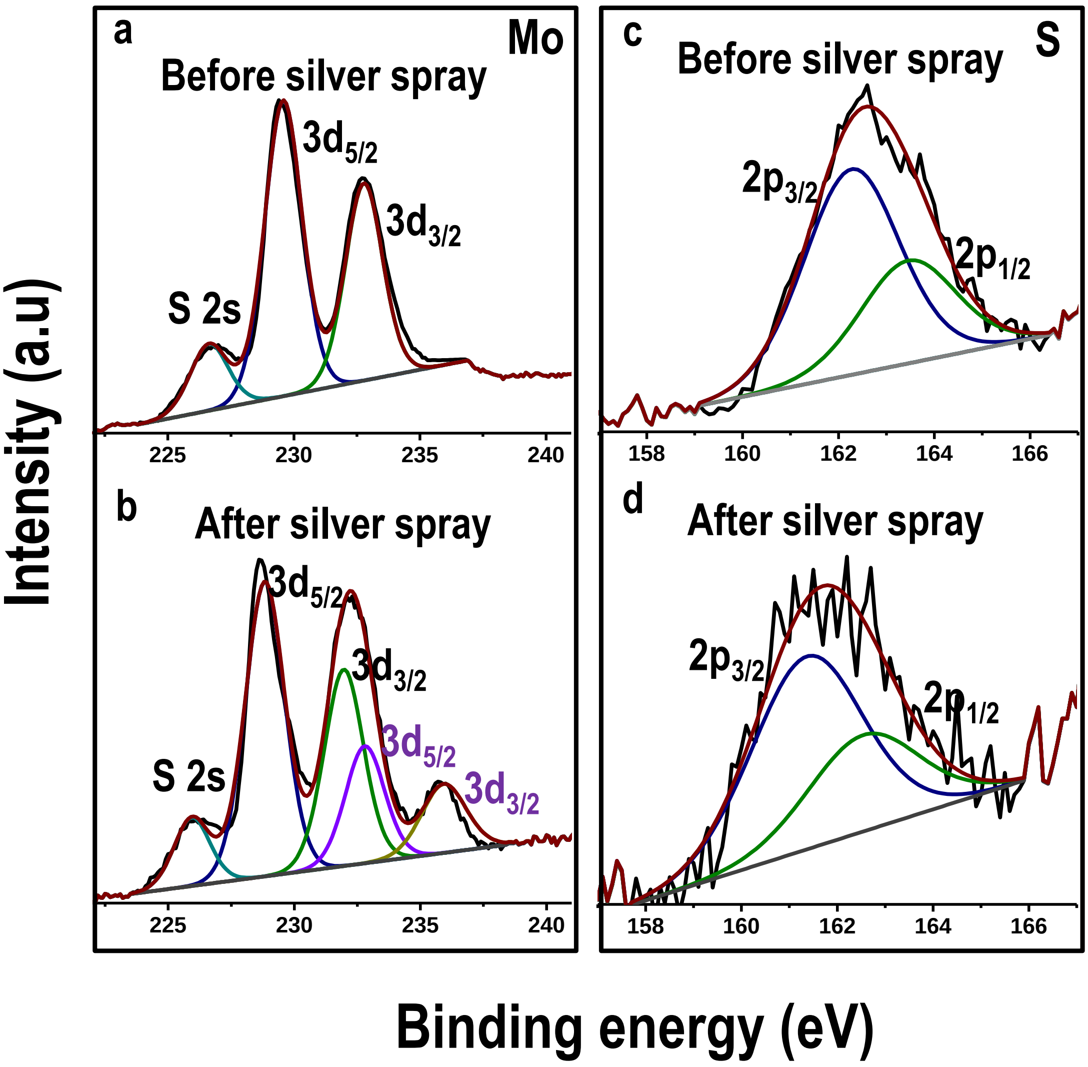

Figure S2. (a-b) correspond to the XPS spectra in the Mo 3d region before and after the electrospray of silver, respectively. (c-d) XPS spectra in the S 2p region before and after the electrospray of silver, respectively.

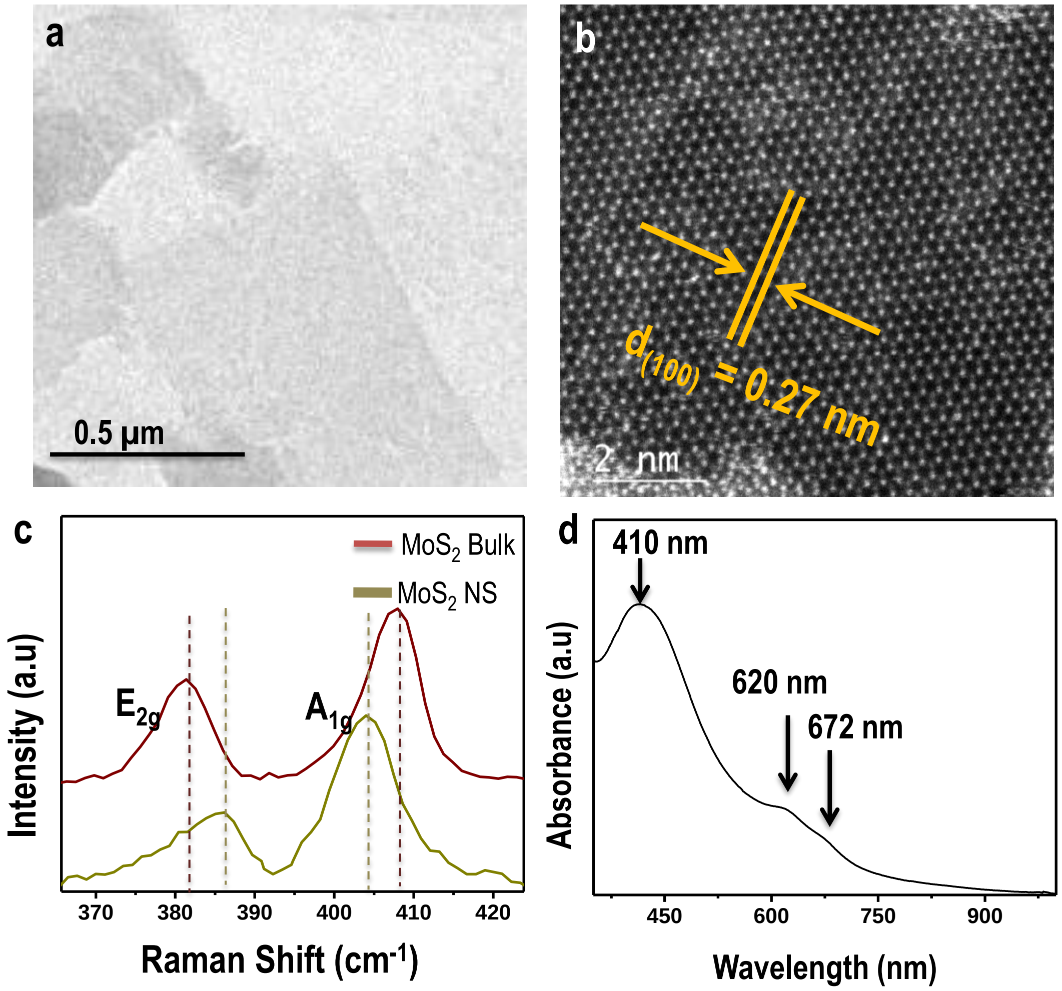

Figure S3. Characterization of MoS_2 NSs. (a) TEM image of as-synthesized MoS_2 nanosheet. (b) HAADF TEM image of (a) MoS_2 nanosheet showing that there are no defects in it. (c) Raman spectrum collected from the MoS_2 nanosheet and bulk MoS_2. The peak difference (~18 cm^{-1}) of E_{2g} and A_{1g} for MoS_2 NSs suggests that the sheets are one layer thick. (d) UV-Vis spectrum collected from a suspension of MoS_2 NSs.

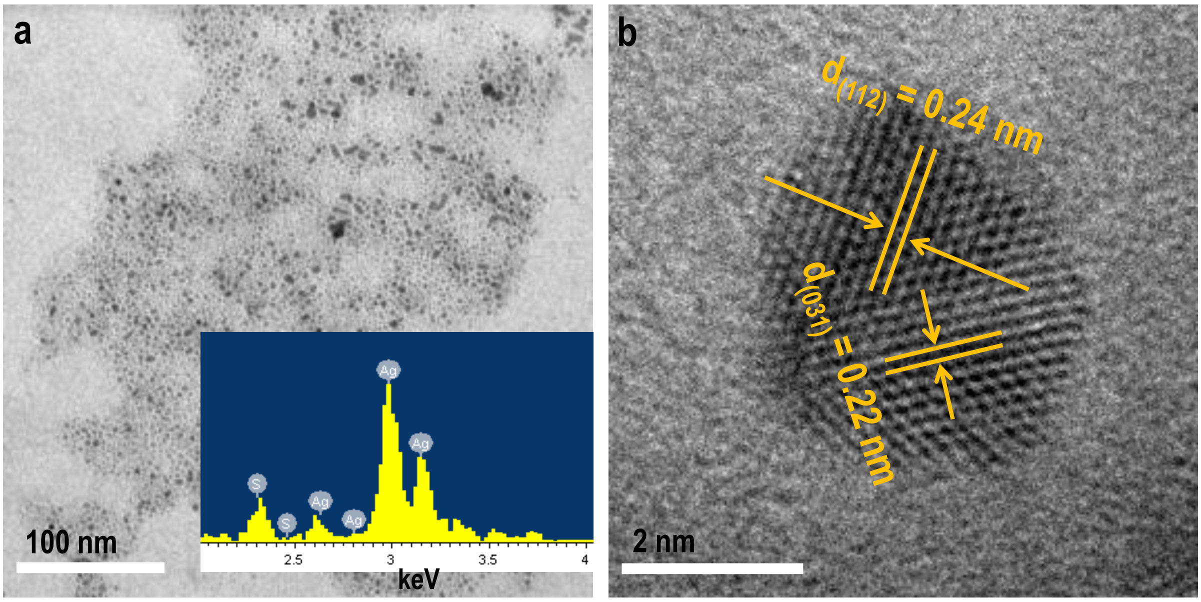

Figure S4. Characterization of Ag_2S NPs. (a) TEM image of the MoS_2 nanosheet showing the complete reaction of it. Inset shows TEM energy dispersive spectrum of Ag_2S particles. (b) HRTEM image of an Ag_2S particle. The d(112) and d(031) planes of 0.24 nm and 0.22 nm suggest that the formed nanoparticles are the acanthite phase of Ag_2S.

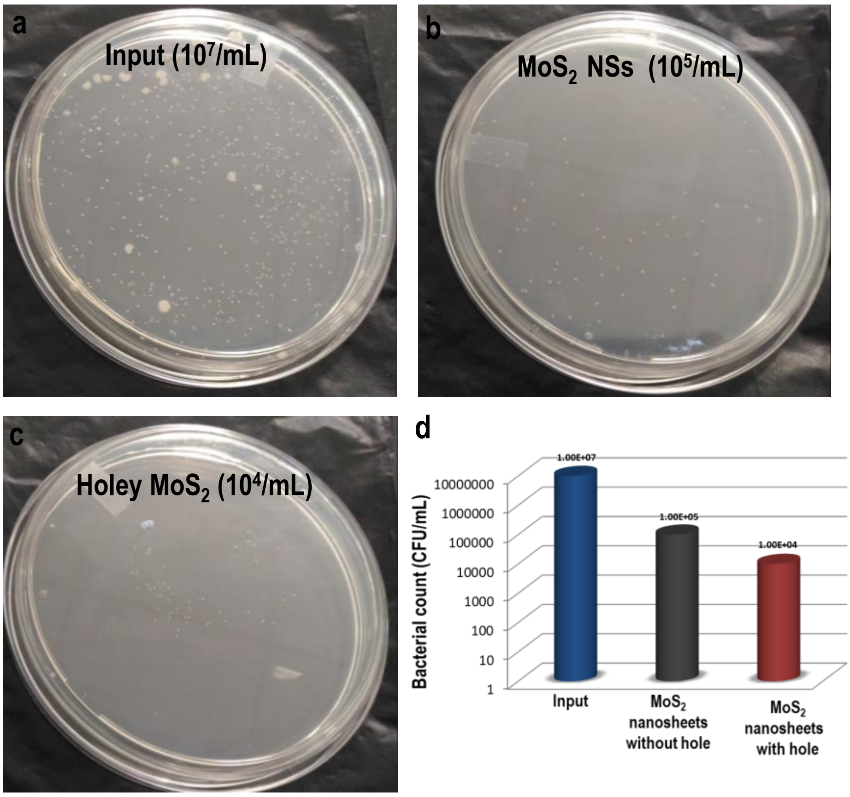

Figure S5. Antibacterial performance of holey MoS_2. (a-c) Photograph of the antibacterial activity (with *Bacillus subtilis* bacteria) of MoS_2 NSs shown in (b) and with holey MoS_2 shown in (c). (a) Input bacterial concentration and (d) comparison of antibacterial activity of MoS_2 NSs and holey MoS_2.

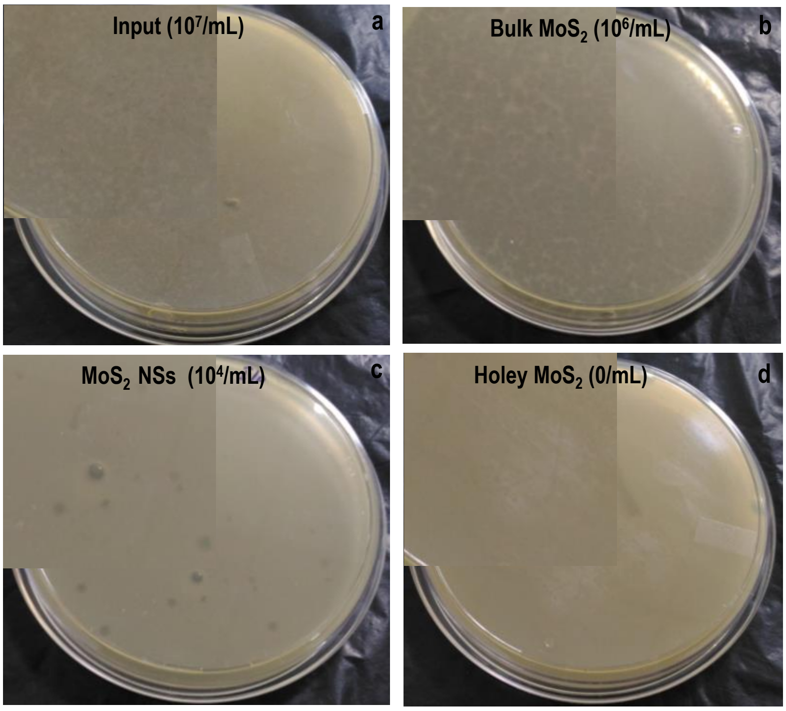

Figure S6. Antiviral activity of holey MoS_2. (a-d) Photograph of antiviral (*bacteriophage MS2*) activity of (b) bulk MoS_2, (c) MoS_2 NSs and (d) holey MoS_2. Expanded views of the petri dishes are shown as insets.

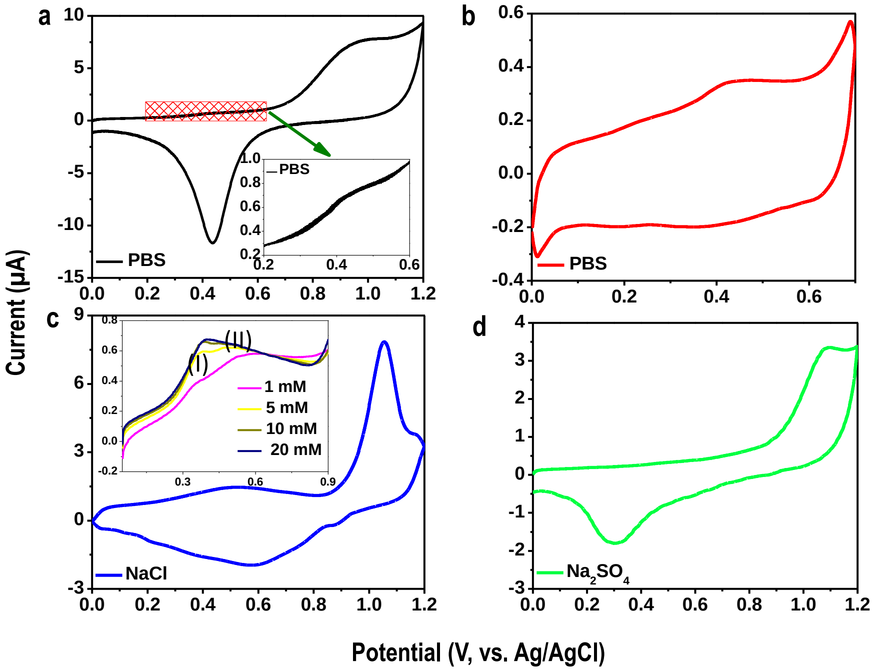

Figure S7. CV traces of Au electrode in different electrolytes. CV traces of gold in (a) PBS; (c) NaCl; (d) Na_2SO_4, respectively. (b) CV trace of gold in PBS scanned up to 0.8 V. Inset of **c** shows the concentration-dependent linear sweep voltammetry (LSV) traces scanned up to 0.9 V.

The CV profile of Au (Figure S7a) measured in PBS exhibits an oxidation peak at +0.95 V and a reduction peak at +0.43 V. These were not seen in the CV measured up to +0.7 V

(Figure S7b) confirming that the oxidation peak at 0.95 V was coupled with the reduction peak at +0.43 V. These were attributed to the reaction, Au + 4Cl$^-$ = AuCl$_4^-$ + 3e$^-$. Although these peaks were not seen in Figure S7b, there was a small oxidation peak at +0.45 V, which is also seen in Figure S7a. In order to understand the origin of this small peak, we performed the same CV measurement (scanned up to +1.2 V) in NaCl solution (Figure S7c) and the peak appeared again at +0.45 V along with the other peaks. However, when we performed the same experiment in Na_2SO_4, the peak around +0.45 V was not seen (Figure S7d). To assign the broad peak around +0.45 V, we performed linear sweep voltammetry (LSV) up to +0.9 V with different concentrations of NaCl (spectra shown in the inset of Figure S7c). Seemingly, the single peak observed at +0.45 V in Figure S7c was actually composed of two peaks as shown in the inset of the same figure. At lower concentrations of NaCl, the two peaks were well separated from each other and the peak separation of these two become narrower as concentration of NaCl was increased. For better clarity, we marked these peaks as I and II. First peak I was due to Cl$^-$ adsorption on Au surface and consecutive oxidation of Au by the reaction, Au$_{(surface)}$Cl$^-$ = AuCl$_{(surface)}$+ e$^-$ (peak II).[2] At lower concentration of NaCl (1 mM), coverage of Cl$^-$ was lower and correspondingly Au oxidation was poor. However, as NaCl concentration was increased, current started increasing. Finally, maximum current was seen at 20 mM NaCl which was due to higher Cl$^-$ coverage and therefore, maximum oxidation. Beyond 20 mM of NaCl concentration, no further change of peak current was observed. As H_2O_2 comes in contact with the electrode ($H_2O_{2(bulk)} \xrightarrow{Diffusion} H_2O_{2(electrode)}$) it gets oxidized ($H_2O_2 = 2H^+ + O_2 + 2e^-$) at the same potential.

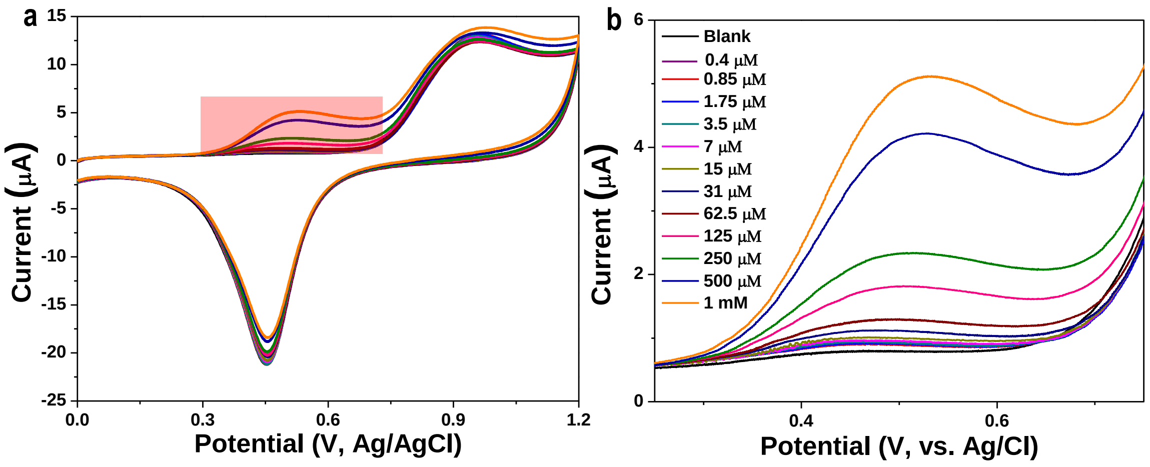

Figure S8. CV of externally added H_2O_2. (a) CV traces of a control experiment performed with externally added H_2O_2 with different concentrations, in PBS. (b) Magnified version of the marked area of Figure S8a.

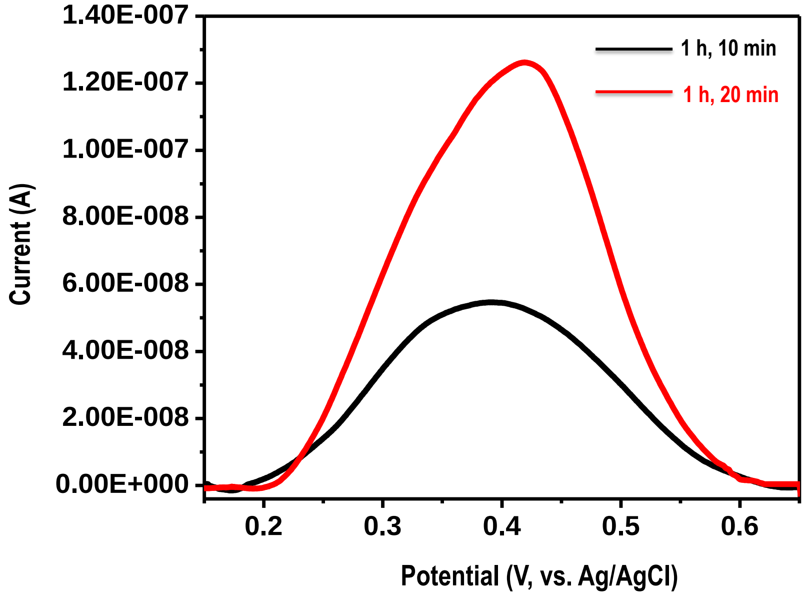

Figure S9. Linear sweep voltammetry (LSV) profile of MoS_2 NS suspensions with different time of spraying of Ag^+. LSV spectrum MoS_2 NSs suspension after 10 and 20 min of Ag^+ deposition and 1h of visible light irradiation.

Samples	Concentration of H_2O_2 (μM)
MoS_2 NSs without hole (1 h)	0.43
MoS_2 NSs without hole (2 h)	0.63
Holey MoS_2 NSs (1 h)	0.69
Holey MoS_2 NSs (1 h)	1.54

Table S1. Quantification of H_2O_2 concentration generated from holey MoS_2 using calibration curves.

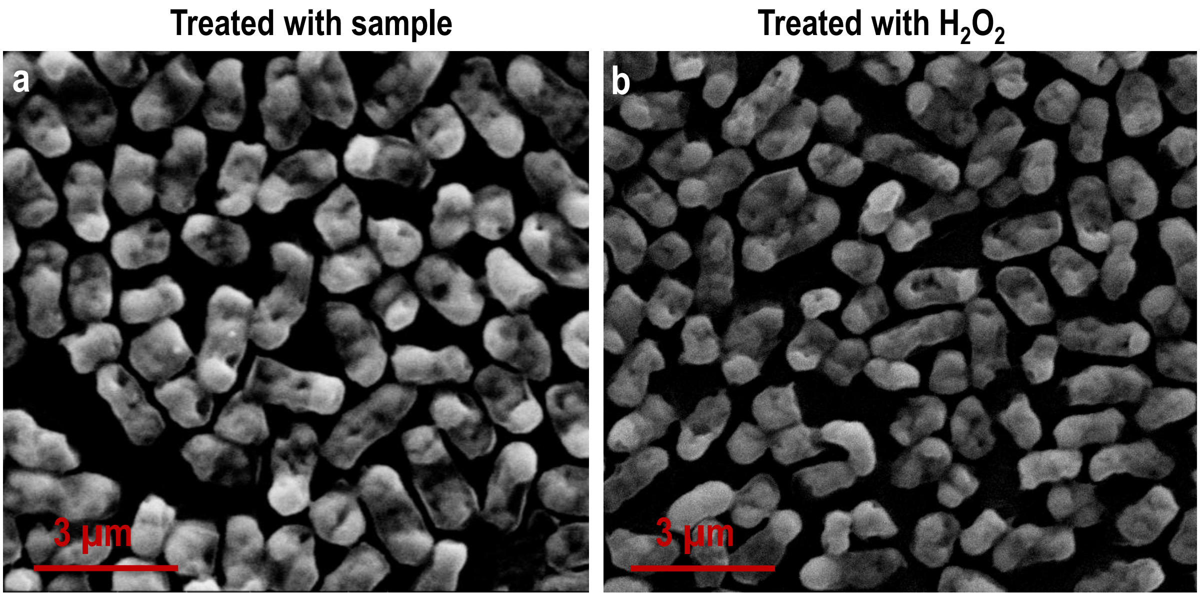

Figure S10. Imaging of dead bacteria. (a-b) SEM images of the bacteria (*E. coli*) after the treatment with the sample and H_2O_2, respectively. In both the cases, similar cell damage was observed.

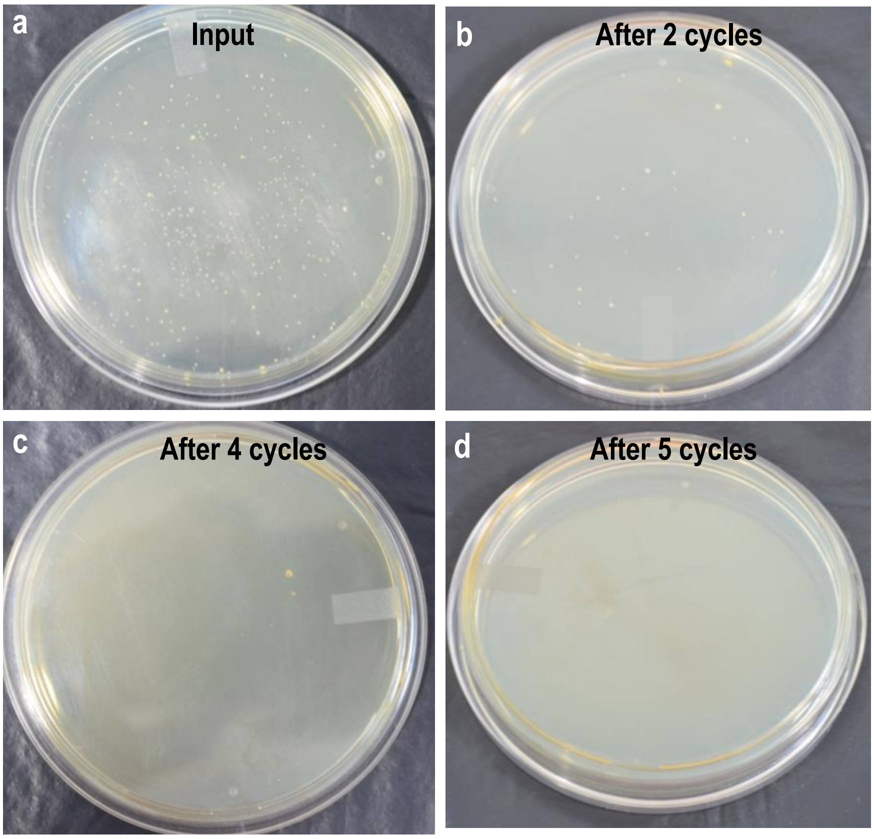

Figure S11. Photographs of the experiment performed with the prototype. (a) Input (contaminated water), bacteria count (b) after 2 cycles, (c) after 4 cycles and (d) after 5 cycles.

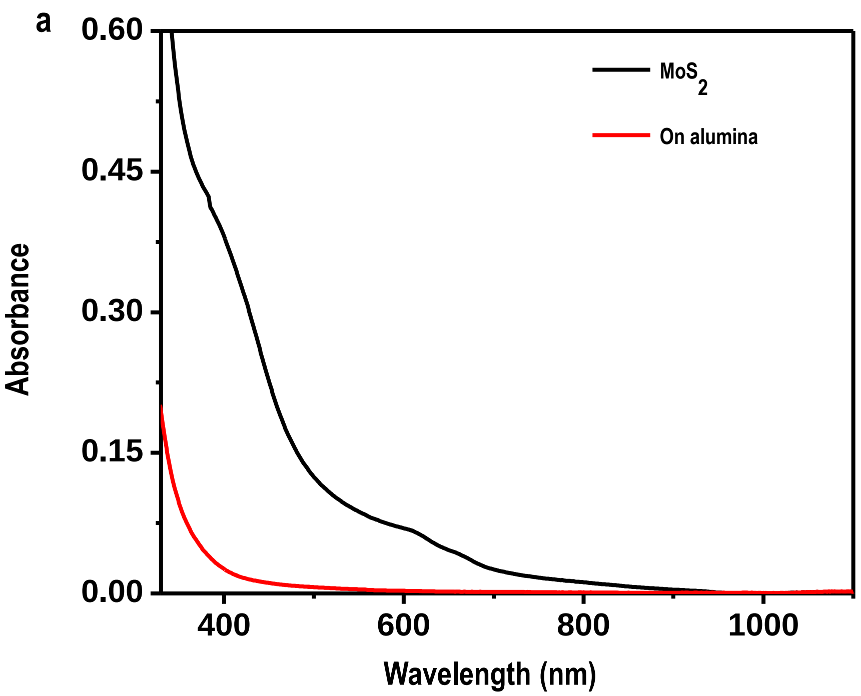

b

Sample	Mo concentration (ppb)
MoS_2 NSs	0.08 ppb

Figure S12. No leaching of MoS_2 from MoS_2-adsorbed alumina. (a) UV-Vis spectra of MoS_2 NSs (black) and the supernatant (red) taken from MoS_2-adsorbed alumina@water. Negligible leaching was observed. (b) ICP MS analysis of the supernatant showed negligible Mo concentration.

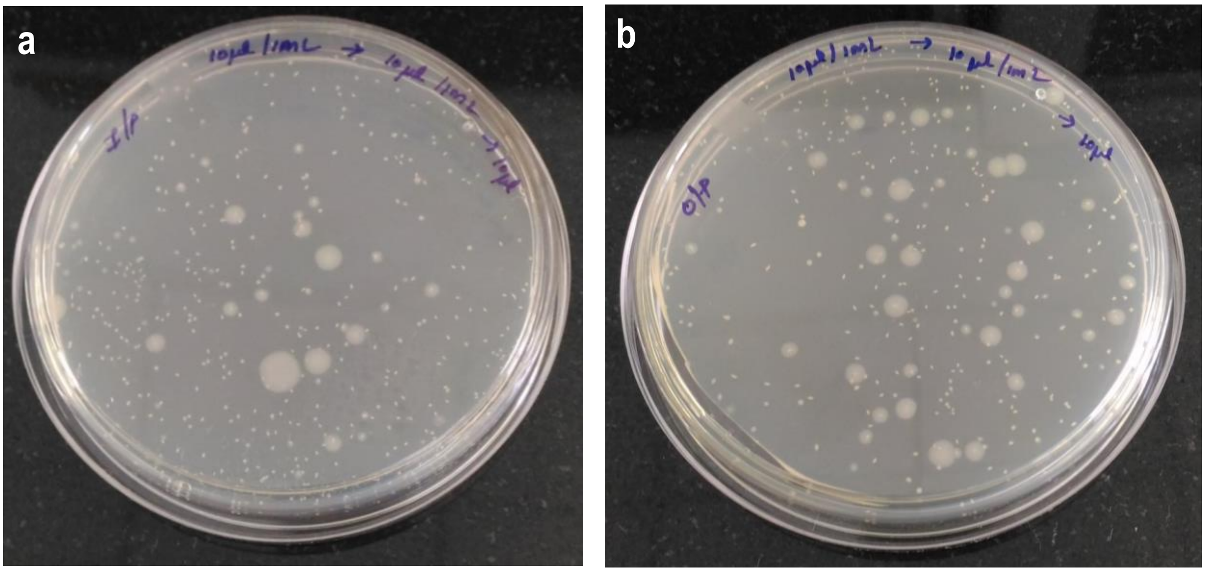

Figure S13. *E. coli* contaminated water was passed through a column packed with only alumina. Photograph of (a) Input (contaminated water) and (b) output. No disinfection was observed.

References

[1] a) L. Yuwen, H. Yu, X. Yang, J. Zhou, Q. Zhang, Y. Zhang, Z. Luo, S. Su, L. Wang, *Chem. Commun. (Cambridge, U. K.)* **2016,** *52*, 529-532; b) L. Yuwen, F. Xu, B. Xue, Z. Luo, Q. Zhang, B. Bao, S. Su, L. Weng, W. Huang, L. Wang, *Nanoscale* **2014,** *6*, 5762-5769; c) C. N. R. Rao, Ramakrishna Matte, S. S. H., U. Maitra, *Angew. Chem., Int. Ed.* **2013,** *52*, 13162-13185.

[2] a) L. Aldous, D. S. Silvester, C. Villagran, W. R. Pitner, R. G. Compton, L. M. Cristina, C. Hardacre, *New Journal of Chemistry* **2006,** *30*, 1576-1583; b) Z. Shi, J. Lipkowski, *J. Electroanal. Chem.* **1996,** *403*, 225-39.
